# Training to Promote Empathic Communication in Graduate Medical Education: A Shared Learning Intervention in Internal Medicine and General Surgery

**DOI:** 10.1089/pmr.2021.0036

**Published:** 2022-03-30

**Authors:** Bethany J. Lockwood, Jillian Gustin, Nicole Verbeck, Kara Rossfeld, Kavitha Norton, Todd Barrett, Richard Potts, Robert Towner-Larsen, Brittany Waterman, Steven Radwany, Christopher Hritz, Sharla Wells-Di Gregorio, Scott Holliday

**Affiliations:** ^1^ Division of Palliative Medicine, The Ohio State University Wexner Medical Center, Columbus, Ohio, USA.; ^2^ Office of Curriculum and Scholarship, The Ohio State University College of Medicine, Columbus, Ohio, USA.; ^3^ Complex General Surgical Oncology, Ohio Health, Columbus, Ohio, USA.; ^4^ Patient Experience, The Ohio State University Wexner Medical Center, Columbus, Ohio, USA.; ^5^ Medical Staff Administration, The Ohio State University Wexner Medical Center, Columbus, Ohio, USA.; ^6^ College Administration, The Ohio State University College of Medicine, Columbus, Ohio, USA.; ^7^ Division of General Internal Medicine, The Ohio State University Wexner Medical Center, Columbus, Ohio, USA.

**Keywords:** communication training, empathy, graduate medical education, shared learning

## Abstract

**Background:**

Empathic communication skills have a growing presence in graduate medical education to empower trainees in serious illness communication.

**Objective:**

Evaluate the impact, feasibility, and acceptability of a shared communication training intervention for residents of different specialties.

**Design:**

A randomized controlled study of standard education v. our empathic communication skills-building intervention: VitalTalk-powered workshop and formative bedside feedback using a validated observable behavioral checklist.

**Setting/Subjects:**

During the 2018–2019 academic year, our intervention was implemented at a large single-academic medical center in the United States involving 149 internal medicine and general surgery residents.

**Measurements:**

Impact outcomes included observable communication skills measured in standardized patient encounters (SPEs), and self-reported communication confidence and burnout collected by surveys. Analyses included descriptive and inferential statistics, including independent and paired *t* tests and multiple regression model to predict post-SPE performance.

**Results:**

Of residents randomized to the intervention, 96% (*n* = 71/74) completed the VitalTalk-powered workshop and 42% (*n* = 30/71) of those residents completed the formative bedside feedback. The intervention demonstrated a 33% increase of observable behaviors (*p* < 0.001) with improvement in all eight skill categories, compared with the control who only showed improvement in five. Intervention residents demonstrated improved confidence in performing all elicited communication skills such as express empathy, elicit values, and manage uncertainty (*p* < 0.001).

**Conclusions:**

Our educational intervention increased residents' confidence and use of essential communication skills. Facilitating a VitalTalk-powered workshop for medical and surgical specialties was feasible and offered a shared learning experience for trainees to benefit from expert palliative care learning outside their field.

## Introduction

Empathy plays a critical role in the delivery of meaningful and effective patient-centered care, cultivating an understanding and promoting validation of patients' experiences. The impact of empathic communication is often evaluated through the lens of the patient. At the same time, it is essential to expand our knowledge of the impact of empathic communication skills on the clinician experience including that of clinician trainees.

A growing body of literature supports efforts to teach empathic communication in graduate medical education (GME) through a variety of techniques with maintenance of skills over time.^1–5^ Nonetheless, there are continued shortcomings in communication between clinicians and patients,^6^ and many clinician trainees feel ill-prepared engaging in difficult discussions such as delivering serious news and talking about dying.^7^ Even more worrisome is the decline in empathy throughout medical training, with particular risk in surgical specialties related to the nature of their technically demanding work and lack of formalized training in empathic communication.^8^ This decline in empathy can reveal depersonalization, an indifferent or impersonal attitude toward others that is often considered a component of burnout. Burnout is highly prevalent in medical careers and empathic communication may serve as a protective factor.^9–11^

One communication teaching framework, VitalTalk serves to assist many specialties in developing effective communication skills for patients with serious illness.^12^ This framework has been evaluated and adapted for a variety of medical residents^13–15^ and fellows,^16–20^ and has demonstrated improved communication skills based on pre- and post-self-assessments,^15,19,20^ family meetings,^17,18^ and standardized patient encounters (SPEs).^13,16^ Trainees also reported increased levels of confidence in their communication skills,^18^ and acceptability of this communication training method.^15,20^

Another teaching tool, formative feedback at the bedside, is ubiquitous in GME and has demonstrated value in improving learner performance and skill acquisition.^21^ In addition, many communication skills have further been operationalized into observable behavioral checklists.^22,23^ Use of a validated behavioral checklist can guide formative bedside feedback and further reinforce learned skills during clinical patient encounters. Thus, a combination of these two teaching methods may enhance knowledge and application to create a robust learning experience.

Although communication training for surgical residents is growing to meet what already exists for medical residents, less is known regarding the potential impact and feasibility of a shared learning experience. This study's unique randomized controlled design and primary outcome of observable communication skills expands the current literature, and implements a communication training intervention in residency programs that historically have limited shared learning at our institution. Our objective is to evaluate the impact, feasibility, and acceptability of a VitalTalk-powered workshop and formative bedside feedback skills-building intervention. We aim to evaluate impact with our primary outcome of observable communication skills measured in SPEs, and secondary outcomes of self-reported communication confidence and burnout measured in pre- and post-intervention surveys.

## Materials and Methods

### Setting and participants

This randomized controlled study was conducted from July 1, 2018 to June 30, 2019 at a large, single-academic medical center in the United States with >800 GME trainees. A total of 149 residents, including internal medicine (*n* = 105) and general surgery (*n* = 44), participated in this pilot program. Within each program all postgraduate year levels were included and residents were randomly assigned to either the standard education control (*n* = 75; 53 medicine, 22 surgery) or the formal communication training intervention (*n* = 74; 52 medicine, 22 surgery).

Randomization was performed by importing residency program rosters to Excel, and using the random selection feature for study assignment. Nongraduating residents in the control group were offered the formal communication training at the completion of the study period to promote equal educational opportunities. This study included both required educational components (e.g., intervention and SPEs) and voluntary research components (e.g., surveys). A waiver of consent applied to the educational components as part of the program curriculum, and informed consent was obtained for the voluntary research components. The Ohio State University Institutional Review Board approved this study (IRB Study #: 2017B0329).

### Curriculum structure and content

Our empathic communication skills-building intervention included two components: (Part 1) VitalTalk-powered workshop^24^ and (Part 2) formative bedside feedback. The eight-hour VitalTalk-powered workshop facilitated by four palliative faculty (B.J.L., J.G., K.N., and T.B.), incorporated didactics, demonstration, and deliberate practice. Didactics served as the foundation and introduced the scaffolding for goals of care communication using REMAP: **R**eframe, **E**xpect emotion, **M**ap values, **A**lign, and **P**lan.

**FIG. 1.**
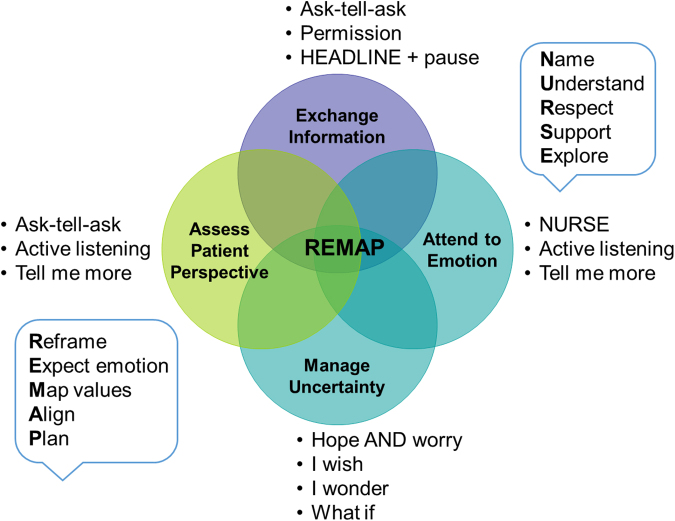
Communication Curriculum Content and Skills. Schema of educational content and skills practiced in VitalTalk-powered workshop and formative bedside feedback encounters.

The majority of the workshop was spent in small group deliberate practice with use of simulated patients, creating opportunities to debrief, brainstorm, and crystallize learning points. Patient cases were designed to be applicable to both specialties and each small group included both medicine and surgery residents to encourage cross-specialty learning and perspectives. Intervention residents completed one full-day workshop and a total of four workshops were facilitated during the study period to accommodate scheduling needs. Figure 1 further depicts a schema of the instructional content and learned skills.

The formative bedside feedback during clinical patient encounters was performed mostly by trained palliative faculty (B.J.L., J.G., K.N., T.B., B.W., S.R., C.H., and S.H.) to reinforce learned skills using the Family Meeting Behavioral Skills Checklist (FMBSC), a 30-item behavioral checklist organized into eight communication skill categories developed at our institution for the purposes of teaching and assessment of physician trainees.^18,25^ The FMBSC (Supplementary File S1) demonstrates internal consistency, structural validity, and inter-rater reliability.^25^

In addition, the skills assessed by the FMBSC are generalizable to a variety of serious illness communication encounters. Skills taught with the VitalTalk framework REMAP can be directly mapped to skills assessed by the FMBSC. For example, **R**eframe with headline: *share clinical information clearly*, **E**xpect emotion and respond with empathy: *acknowledge distress (*e.g., *name emotion)* and *validate reactions with empathic statements*, and **M**ap and **A**lign values before recommending a **P**lan: *focus on patient's values before discussion of treatment options* and *offer a recommendation in keeping with preferences*.

The control group received standard education including one formal 20-minute small group workshop during GME orientation intern year and informal discussions that may have occurred during routine patient care. Both groups participated in pre- and post-videotaped SPEs that offered additional learning opportunities with optional resident self-review and reflection (only two residents pursued this). Each SPE experience consisted of a 20-minute session with a standardized patient (12 SPs trained by B.J.L.) who portrayed the role of a spouse of an unresponsive patient in the medical or surgical intensive care unit (ICU). Although details of the pre- and post-SPE cases were slightly different to avoid habituation, there were consistent themes (e.g., unresponsive patient with significant neurological injury on mechanical ventilation) and tasks to deliver serious news and explore goals of care.

### Outcomes measured

Our primary impact outcome was the use of observable communication skills measured in SPEs, and secondary outcomes of self-reported communication confidence and burnout measured in pre- and post-intervention surveys. Complete study measures are illustrated in Table 1.

**Table 1.** Empathic Communication Training Study Measures

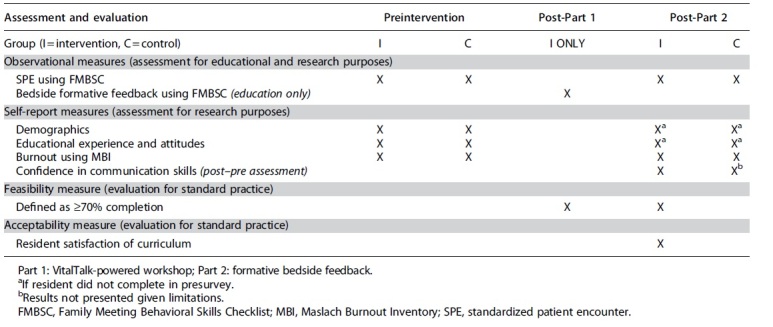

#### Observational measures

For the intervention and control groups, a required videotaped SPE scored by trained faculty (B.J.L., K.R., R.P., and R.T.L.) using the FMBSC provided an observational pre- and postmeasure approximately eight months apart. The number of observed behaviors out of 30 total was tallied and converted to a percentage score.^18^ Faculty raters completed an observer training session and represented diverse backgrounds, including palliative medicine, general surgery, patient experience, and professional coaching. The formative bedside feedback using the FMBSC in clinical patient encounters was for educational purposes only as part of the intervention curriculum and not included as a research measure.

#### Self-report measures

Preassessment for both groups utilized a voluntary electronic survey administered by Qualtrics assessing demographics, prior educational experience and attitudes, and burnout using the Maslach Burnout Inventory (MBI),^26^ specifically the MBI Human Services Survey for Medical Personnel.^27^ At approximately eight months following, postassessment for both groups included a voluntary electronic survey administering the same demographics if not previously completed (to capture largest sample), MBI, and a self-report of communication confidence as a post–pre assessment. A post–pre assessment collects data at the same time point (e.g., at conclusion of training or study), asking participants to rate their confidence before (retrospectively) and after the intervention or designated time period, and can offer a more sensitive measure of effects and minimize response-shift bias.^28–30^

#### Feasibility and acceptability of communication training intervention

Intervention feasibility was defined as ≥70% completion, and reported for each component of the two-part intervention to determine if one and/or both parts were feasible. Acceptability was assessed by participation feedback included in the intervention group voluntary postsurvey.

### Data analysis

#### Descriptive

Descriptive statistics were used to report demographics, prior educational experience and attitudes, mean MBI subscale scores (emotional exhaustion, depersonalization, and personal accomplishment),^27^ and mean SPE scores using the FMBSC.

#### Inferential

A paired *t* test was used to evaluate pre- and postperformance of observable communication skills in SPEs measured by the FMBSC and eight subscales: (1) assess perception, (2) elicit communication preferences, (3) exchange clinical information, (4) assess/attend to reactions, (5) manage uncertainty, (6) share decision making, (7) summarize/plan, and (8) general approach.^25^ A paired *t* test was also used to evaluate changes in communication confidence, comparing pre- and postratings within a study group. An independent/two-sample *t* test was attempted to compare confidence between study groups, however was limited due to low response rate for control group. Finally, a multiple regression analysis was performed to assess the predictive value of independent variables, including pre-SPE score, residency program, and study assignment (control vs. intervention) on the dependent variable post-SPE score (at the FMBSC total and subscale level). IBM SPSS Statistics version 26 was used for all statistical analyses.

## Results

### Demographic characteristics of participants

The majority of participant respondents were aged 25 to 29 years (61%), male (53%), and White (62%) with postresidency plans for fellowship training (59%). Resident demographic characteristics are further summarized in Table 2.

**Table 2.** Resident Demographic Characteristics

	Overall (*n* = 66), *n* (%)^a^
Age (years)	
20–24	1 (1.5)
25–29	40 (60.6)
30–34	20 (30.3)
35–39	3 (4.5)
Missing	2 (3.0)
Gender	
Female	29 (43.9)
Male	35 (53.0)
Missing	2 (3.0)
Race	
White	41 (62.1)
Black	6 (9.1)
Asian	11 (16.7)
Prefer not to answer	5 (7.6)
Multiple	1 (1.5)
Missing	2 (3.0)
Postresidency plans	
Academic center	16 (25.0)
Community hospital	5 (7.8)
Outpatient clinic	5 (7.8)
Fellowship training	38 (59.4)
Missing	2 (3.0)

^a^ Obtained from either pre- or postsurvey to capture largest sample. Participant selected gender and race shown here, other identifying options available in survey.

### Measure completion rates and feasibility of intervention

For the entire cohort of 149 residents, 84% (*n* = 125/149) completed the pre-SPE and 72% (*n* = 90/125) of those residents completed the post-SPE assessment. Total voluntary electronic survey response rates were low even with up to three reminders at 34% (*n* = 50/149) pre- and 27% (*n* = 40/149) post. Of the 74 residents randomly assigned to the intervention, 96% (*n* = 71/74) completed the VitalTalk-powered workshop and 42% (*n* = 30/71) of those residents also completed the formative bedside feedback.

### Prior educational experience and attitudes

Of respondents from both study groups, many reported prior communication training experience through different teaching methods as depicted in Table 3. The residents rated methods of role play and/or simulated patient as most helpful, followed by small group and lecture, respectively. Residents had variable prior experience with specific communication techniques. The instruction of empathic communication skills during residency training was identified by 94% (*n* = 47/50) of respondents as important with 66% (*n* = 33/50) rating this extremely important.

**Table 3.** Prior Communication Training Experience: Methods and Skills

Type of training experience	*n* (%^v^)	Training through modeled behavior	*n* (%^v^)
Lecture series	42 (79.2)	By residents	41 (78.8)
Small group discussion	43 (84.3)	By fellows	34 (68.0)
Role play/simulated patient	48 (90.6)	By attendings	43 (84.3)
Other	2 (14.3)	By other	9 (32.1)

Communication content taught how to…	*n* (%^v^)	Communication skills and techniques	*n* (%^v^)
Elicit perceptions of illness	48 (94.1)	Ask-Tell-Ask	35 (70.0)
Deliver bad news	48 (94.1)	Tell me more	48 (96.0)
Express empathy	49 (96.1)	Active listening	48 (96.0)
Elicit hopes/fears about illness	45 (88.2)	NURSE mnemonic	24 (48.0)
Promote prognostic awareness	38 (74.5)	Hope and worry	27 (54.0)
Manage uncertainty	33 (64.7)	I wish statements	25 (50.0)
Elicit values, goals, and preferences	45 (88.2)	Reframing why status quo not working	28 (56.0)
Recommend treatment plan	44 (86.3)	Map the future	22 (44.0)
		Align with patient's values	44 (88.0)

Overall *n* = 66; actual *n* and valid percentage (%^v^) reported to account for respondent attrition at the item level.

NURSE, Name Understand Respect Support Explore.

### Communication skill use

Ninety residents (60% of total 149) completed both pre- and post-videotaped SPEs for skill assessment using the FMBSC. Residents regardless of program or study assignment had a significant improvement over time (*p* < 0.001), with an overall increase in mean observed communication behaviors from 56% (16.91/30 behaviors) pre- to 72% (21.54/30 behaviors) post as shown in Table 4. The intervention residents demonstrated a 33% increase in observable behaviors (*p* < 0.001) on the post-SPE (Table 4).

**Table 4.** Observable Communication Skills in Standardized Patient Encounters

	*n*		Mean % of FMBSC observed behaviors	SD		*p*
Paired pre- and post-SPE scores: total						
Pre	90		56.07	15.77		<0.001
Post	90		72.04	20.31		
Paired pre- and post-SPE scores: control						
Pre	45		57.26	16.61		0.003
Post	45		70.81	22.09		
Paired pre- and post-SPE scores: intervention						
Pre	45		54.89	14.97		<0.001
Post	45		73.26	18.54		

Paired FMBSC subscales: skill categories						
	Control (*n* = 45)			Intervention (*n* = 45)		
	*t*	*p*		*t*	*p*	
Assess perception	2.211	0.032	^*^	3.292	0.002	^**^
Elicit communication preferences	1.709	0.095		3.309	0.002	^**^
Exchange clinical information	3.362	0.002	^**^	3.413	0.001	^***^
Assess/attend to reactions	1.416	0.164		2.223	0.031	^*^
Manage uncertainty	3.511	0.001	^***^	5.477	<0.001	^***^
Share decision making	0.797	0.430		2.899	0.006	^**^
Summarize/plan	2.953	0.005	^**^	3.084	0.004	^**^
General approach	2.789	0.008	^**^	5.216	<0.001	^***^

Absolute *t*-values reported from paired *t* test comparing pre- and post-SPE subscale scores, *df* = 44.

^*^ *p* < 0.05; ^**^p < 0.01; ^***^p ≤ 0.001; significance (two-tailed).

SD, standard deviation.

Further analysis of the eight FMBSC subscales (skill categories) represented in Table 4 (bottom) offers a comparison of pre- and post-SPE scores at the skill category level for each study group with visual representation of various significance levels. Intervention residents demonstrated significant improvement in all eight subscales on the post-SPE (*p* range <0.001 to 0.031) including three skill categories that the control group did not: *elicit communication preferences*, *assess/attend to reactions*, and *share decision making* (Table 4). An additional report is available illustrating changes in observed behaviors by residency program (Supplementary File S2).

A multiple regression analysis showed no significant predictive value of the pre-SPE score, residency program, or study assignment on the post-SPE performance, with consistent results when evaluating at the FMBSC total and subscale levels with a single exception. For the subscale *elicit communication preferences*, the results indicated a significant coefficient for the pre-SPE subscale score (*p* < 0.001), indicating that pre-SPE performance was significantly predictive of post-SPE performance on this subscale, *F*(3, 86) = 7.526, *p* < 0.001 (Supplementary File S3).

### Communication confidence and burnout

Participating intervention residents reported a significant improvement (*p* < 0.001) in communication confidence across all skills measured by the post–pre confidence assessment as shown in Table 5. Results are not reported for the control group due to low response rate. In review of pre- and post-MBI subscale scores (emotional exhaustion, depersonalization, and personal accomplishment), respondents had consistent levels of reported burnout irrespective of study assignment or program. Mean MBI pretest scores of 23.4 (SD 10.22), 11.0 (SD 7.03), and 37.9 (SD 7.42), suggested moderate emotional exhaustion, high depersonalization, and moderate personal accomplishment based on commonly used MBI cutoff scores, although substantial variability exists and cutoffs have further been removed from the most recent MBI version.^27,31^

**Table 5.** Intervention Residents' Confidence in Communication Skills

Pre, mean (SD)	Post, mean (SD)	*p*	Skill
3.04 (0.85)	4.07 (0.68)	<0.001	Assess patient perspective of illness experience
3.21 (1.10)	4.43 (0.63)	<0.001	Give serious news to a patient about his/her illness
3.54 (0.79)	4.39 (0.74)	<0.001	Express empathy
2.93 (0.90)	3.93 (0.77)	<0.001	Respond to patients who deny seriousness of illness
3.04 (0.96)	4.14 (0.80)	<0.001	Elicit patient's hopes and fears about serious illness
3.14 (1.15)	4.18 (0.72)	<0.001	Promote prognostic awareness related to illness
2.93 (0.94)	3.89 (0.79)	<0.001	Manage uncertainty
3.11 (0.85)	4.15 (0.82)	<0.001	Elicit patient's values, goals, and preferences
3.29 (0.98)	4.39 (0.57)	<0.001	Discuss and recommend a treatment plan

Obtained as a post–pre assessment administered in postsurvey after completion of the intervention. Sample limited to respondents of postsurvey, *n* = 28. Scale example: 1. Not well prepared; 3. Somewhat prepared; 5. Very well prepared.

### Acceptability of training experience

Feedback was obtained as part of the electronic postsurvey for participating intervention residents, with a lower response rate of 23% (*n* = 16/71) for this section. Of respondents, 94% (*n* = 15) identified this training to have good to excellent relevance and good to excellent quality of the workshop. Bedside feedback sessions were rated similarly with good to excellent effectiveness. The majority of respondents (81%, *n* = 13) recommended this training and suggested it even be required.

Additional resident feedback themes for program strength included practical scenarios with simulated patients, real-time feedback, active and engaging small group practice, applicable for broad settings, and encouraged the development of empathy. Themes for program improvement included eliminate bedside feedback component, shorten workshop length, more reinforcement of learned skills at a later time, scheduling interfered with clinical duties, and need to expand to all trainees (e.g., similar to Advanced Cardiovascular Life Support [ACLS] training).

## Discussion

Our communication training intervention demonstrated a significant improvement in all skill categories, including a highly significant difference in many of the subscales, further elevating the intervention's impact of these observable behaviors. Compared with the control group, intervention residents showed significant improvement in three specific skill categories that can be mapped to the intervention's workshop curriculum: *elicit communication preferences*, *assess/attend to reactions*, and *share decision making*.

The intervention enhanced these skills with a curriculum focus on techniques, including ask-tell-ask to elicit preferences, Name Understand Respect Support Explore to attend to reactions, and REMAP as a framework for shared decision making. For example, the intervention's improvement in the *share decision making* category on the FMBSC includes the following skills: achieved common understanding of patient's condition (**R**eframe), focused discussion on goals/values before discussion of interventions (**M**ap and **A**lign values), discussed treatment options based on goals/values and offered recommendations when keeping with decision-making preferences (**P**lan).

Many factors likely influenced the finding that all residents improved over time, including the increased palliative penetrance at our institution of 9.7%, compared with the mean penetration (5.6%) reported of adult hospitals.^32^ Owing to higher penetrance, learners have multiple touchpoints with palliative clinicians who often offer teaching regarding communication. Furthermore, internal medicine residents at our institution have additional learning opportunities including lectures that may incorporate communication pearls and participation in a palliative elective (e.g., 14 medicine residents, 7 who were assigned to the control group, completed a palliative elective during the study period). Finally, merely emphasizing the curricular importance of empathic communication may also influence the significant improvement in skills over time, such that the value placed on a learned skill impacts the residents' desire to master it and is thus reflected in behavior.

Using our feasibility threshold of 70% completion, the mixed medical and surgical VitalTalk-powered workshop was feasible, whereas the formative bedside feedback was not. Formative feedback feasibility was challenged by resident rotation schedule, unexpected patient care needs, and resident buy-in. Given the robust autonomy at our institution, residents' experience of formalized direct observation is variable and perhaps even waning, highlighting an institutional cultural opportunity to bring the clinical transaction back to the bedside to overcome these barriers.

### Limitations

This study represents an educational intervention at a large single academic medical center, targeting ∼70 trainees thus generalizability may be limited for other center types and sizes. Our self-report measures of communication confidence and burnout were limited due to low response rates using voluntary electronic surveys. Given variability in response rates among study groups, at times conclusions could only be descriptive or drawn from within rather than between groups. In addition with our design, certain measures were only obtained for the intervention group limiting comparison based on study assignment and thus not reported (e.g., patient perception).

This could be overcome with design changes to collect all measures for both groups. Our acceptability assessed by participation feedback included in the voluntary postsurvey was similarly limited by low response rate. This could potentially be overcome by postsurvey administration either written or electronic while in-person at intervention close. Finally, with a two-part intervention there are additional limitations including potential for dropout (e.g., 71 residents completed the VitalTalk-powered workshop, yet only 30 completed the bedside feedback) and in identifying which part of the intervention provided the greatest impact.

### Future considerations

With the evolving impact of the COVID-19 pandemic, future adaptations should include a condensed virtual workshop format when needed, which may allow a larger reach to other training programs and institutions, increasing the sample size and ability to further analyze the intervention's impact. Successful formative feedback would also require additional training of primary residency faculty to familiarize use of the FMBSC and expand use beyond palliative faculty to create more opportunities for assessment and improvement in feasibility.

## Conclusions

Although other studies have explored benefits of communication training with the VitalTalk method, this study offers a unique collaborative learning experience for medical and surgical residents. The workshop component of this two-part intervention is feasible and effective demonstrated by our primary outcome of improved observable communication skills and secondary outcome of improved confidence. Our intervention expanded communication training to other specialties and training levels not historically trained at our institution with significant resident-focused benefits to empower serious illness communication.

## Clinical Palliative Care Program

### Program structure

Our palliative care (PC) program is sponsored by our academic hospital system with a payment structure of fee-for-service and small proportion of philanthropy.

### Team staffing

For our entire hospital system, our dedicated PC team includes physician 17.25 full time equivalent (FTE), advanced practice provider 14.6 FTE, clinical psychologist 1.0 FTE, registered nurse 2.5 FTE, social worker 3.0 FTE, and pharmacist 5.0 FTE.

### Program availability

Inpatient PC consultation services are available seven days a week at our main medical center and cancer hospital, with reduced staffing on weekends. Inpatient PC consultation at our community hospital is limited to five days per week in the ICU only. Ambulatory PC services are available five days a week for cancer patients and two days a week for cardiac patients, with phone triage available after hours and weekends. We have a variety of dedicated clinics for specialized populations. We do not provide an inpatient admitting service, hospice service, or home-based PC service.

### Patient volume and interactions

Our average inpatient daily census is 60 patients. Across our health system we average 12,900 inpatient and 6700 outpatient billed PC encounters per year.

## Supplementary Material

Supplemental data

Supplemental data

Supplemental data

## Abbreviations Used

FMBSC: Family Meeting Behavioral Skills Checklist
GME: graduate medical education
ICU: intensive care unit
MBI: Maslach Burnout Inventory
NURSE: Name Understand Respect Support Explore
SD: standard deviation
SPE: standardized patient encounter
