# Supplementary material for: Training to Promote Empathic Communication in Graduate Medical Education: A Shared Learning Intervention in Internal Medicine and General Surgery
Source: Palliat Med Rep. 2022 Mar 30;3(1):26–35. doi: 10.1089/pmr.2021.0036 (PMC8994435; doi:10.1089/pmr.2021.0036)
Supplement: Supplemental data [file Suppl_FileS1.pdf]

Supplemental File 1. FMBSC

Trainee Name: _ Date:_
Observer: _

Family Meeting Behavioral Skills Checklist

**Behavioral skills checklist**	**Behavior performed**		
**Assess/Understand family and patient perception**	Yes	No	n/a
Expressed interest in patient as a person, prior to illness	□	□	□
Asked pt/family to share perspective on patient's illness e.g. ask-tell-ask	□	□	□
**Elicit pt/family preferences for communication**	Yes	No	n/a
Asked pt/family who the team should contact regarding patient condition	□	□	□
Asked pt/family about amount of detail that would be helpful re: clinical condition	□	□	□
Explored pt/family's decision-making preferences	□	□	□
**Exchange/Share clinical information with pt/family**	Yes	No	n/a
Clearly stated the patient's clinical condition with avoidance of medical jargon	□	□	□
Asked if the pt/family understood the information conveyed e.g. ask-tell-ask	□	□	□
Gave "warning" of difficult news with time for preparation	□	□	□
Provided information in short "chunks"	□	□	□
Provided level of detail to match pt/family's desired level of detail	□	□	□
**Assessing/Attending to patient and family reactions**	Yes	No	n/a
Explored pt/family's psychosocial and emotional concerns	□	□	□
Explored pt/family's spiritual and cultural concerns	□	□	□
Acknowledged/accurately reflected pt/family's distress e.g. name emotion	□	□	□
Validated pt/family reaction e.g. empathic statement	□	□	□
**Manage uncertainty**	Yes	No	n/a
Assessed prognostic awareness e.g. hope/ worry	□	□	□
Identified uncertainty of patient's clinical trajectory e.g. hope/worry	□	□	□
Promoted normative coping e.g. hope for best/prepare for worst	□	□	□
**Share decision-making**	Yes	No	n/a
Achieved common understanding of patient's clinical condition	□	□	□
Focused discussion on patient values/goals prior to discussion of specific interventions	□	□	□
Discussed treatment options based on patient's goals/values	□	□	□
Offered recommendations when in keeping with pt/family's decision-making preferences	□	□	□
**Summarize/Plan**	Yes	No	n/a
Summarized discussion	□	□	□
Suggested next steps including future contact between pt/family and care team	□	□	□
Prepared pt/family for the unexpected	□	□	□
Provided necessary resources/contact information to help support pt/family	□	□	□
**General approach**	Yes	No	n/a
Used reflective questioning	□	□	□
Invited pt/family questions throughout meeting	□	□	□
Listened without interruption	□	□	□
Allowed silence	□	□	□
Demonstrated non-verbal cues of empathy/engagement	□	□	□

Authors: J Gustin MD, J McCallister MD, S Wells-DiGregorio PhD, Dave Way MEd

© 2009 The Ohio State University. This work may be reproduced and redistributed, in whole or in part, without alteration and without prior written permission, solely by educational institutions for nonprofit administrative or educational purposes provided all copies contain the following statement: "© 2009 The Ohio State University. This work is reproduced and distributed with the permission of The Ohio State University. No other use is permitted without the express prior written permission of The Ohio State University. For permission, contact J Gustin, MD (jillian.gustin@osumc.edu) or J McCallister MD (Jennifer.mccallister@osumc.edu).
