# Supplementary material for: Training to Promote Empathic Communication in Graduate Medical Education: A Shared Learning Intervention in Internal Medicine and General Surgery
Source: Palliat Med Rep. 2022 Mar 30;3(1):26–35. doi: 10.1089/pmr.2021.0036 (PMC8994435; doi:10.1089/pmr.2021.0036)
Supplement: Supplemental data [file Suppl_FileS2.docx]

Supplemental File 2. Observable Communication Skills in Standardized Patient Encounters by Residency Program

|  | **n** | **Mean % of FMBSC Observed Behaviors** | **SD** | **p-value** |
| --- | --- | --- | --- | --- |
| **Pre-SPE Score** | | | | |
| Medicine | 93 | 54.77 | 17.54 | 0.686 |
| Surgery | 32 | 56.15 | 13.42 |  |
| **Post-SPE Score** | | | | |
| Medicine | 66 | 72.27 | 20.88 | 0.856 |
| Surgery | 24 | 71.39 | 19.08 |  |
| **Paired FMBSC Subscales: Skill Categories** | | | | |
| \|  \| **Medicine**  (n = 66) \| \| \| **Surgery**  (n = 24) \| \| \| \| --- \| --- \| --- \| --- \| --- \| --- \| --- \| \|  \| *t* \| *p* \| \| *t* \| *p* \| \| \| Assess perception \| 3.557 \| 0.001 \| *** \| 1.282 \| 0.213 \|  \| \| Elicit communication preferences \| 3.074 \| 0.003 \| ** \| 1.592 \| 0.125 \|  \| \| Exchange clinical information \| 4.579 \| <0.001 \| *** \| 1.827 \| 0.081 \|  \| \| Assess/Attend to reactions \| 1.828 \| 0.072 \|  \| 2.077 \| 0.049 \| * \| \| Manage uncertainty \| 5.572 \| <0.001 \| *** \| 2.642 \| 0.015 \| * \| \| Share decision-making \| 1.965 \| 0.054 \|  \| 1.629 \| 0.117 \|  \| \| Summarize/Plan \| 3.548 \| 0.001 \| *** \| 2.379 \| 0.026 \| * \| \| General approach \| 4.953 \| <0.001 \| *** \| 2.480 \| 0.021 \| * \| | | | | |

Absolute t-values reported from paired t-test comparing pre and post-SPE subscale scores, df = 65 for medicine, df = 23 for surgery

* p < 0.05; ** p < 0.01; *** p ≤ 0.001; Significance (2-tailed)

Abbreviations: FMBSC, Family Meeting Behavioral Skills Checklist; SD, standard deviation; SPE, standardized patient encounter
