# Supplementary material for: Training to Promote Empathic Communication in Graduate Medical Education: A Shared Learning Intervention in Internal Medicine and General Surgery
Source: Palliat Med Rep. 2022 Mar 30;3(1):26–35. doi: 10.1089/pmr.2021.0036 (PMC8994435; doi:10.1089/pmr.2021.0036)
Supplement: Supplemental data [file Suppl_FileS3.docx]

Supplemental File 3. Additional Analyses – Multiple Regression

**Analysis of Covariance**

Independent variable: study assignment

Covariate: residency program

Dependent variable: total post-SPE score

Levene’s Test of Equality of Error Variances: F (1, 88) = 2.739, p = 0.101

| **Source** | **ANCOVA Between-Subjects Effects** |
| --- | --- |
| Study assignment | F (1, 86) = 0.311, p = 0.578, η^2^ = 0.004 |
| Residency program | F (1, 86) = 0.175, p = 0.677, η^2^ = 0.002 |
| Study assignment * residency program | F (1, 86) = 0.188, p = 0.666, η^2^ = 0.002 |

R^2^ = 0.007 (Adjusted R^2^ = -0.028)

**Multiple Regression Summary**

Independent variables: subscale pre-SPE score, residency program, study assignment

Dependent variable: subscale post-SPE score

| **FMBSC Subscale** | **Model Summary (R^2^)** | **ANOVA** | **Coefficients** |
| --- | --- | --- | --- |
| 1 | 0.043 | F (3, 86) = 1.277, p = 0.287 | None significant |
| 2 | 0.208 | F (3, 86) = 7.526, p < 0.001 | Pre-SPE score, p < 0.001 |
| 3 | 0.026 | F (3, 86) = 0.772, p = 0.513 | None significant |
| 4 | 0.001 | F (3, 86) = 0.032, p = 0.992 | None significant |
| 5 | 0.011 | F (3, 86) = 0.323, p = 0.809 | None significant |
| 6 | 0.047 | F (3, 86) = 1.406, p = 0.246 | None significant |
| 7 | 0.021 | F (3, 86) = 0.605, p = 0.614 | None significant |
| 8 | 0.067 | F (3, 86) = 2.066, p = 0.111 | Study assignment, p = 0.075* |

* approaching significance

FMBSC Subscales

1: Assess perception

2: Elicit communication preferences

3: Exchange clinical information

4: Assess/Attend to reactions

5: Manage uncertainty

6: Share decision-making

7: Summarize/Plan

8: General approach
